# A rare case of anasarca caused by infiltration of the pituitary gland by diffuse large B-cell lymphoma

**DOI:** 10.1186/s12902-015-0007-4

**Published:** 2015-03-25

**Authors:** Ayako Kumabe, Tsuneaki Kenzaka, Yoshioki Nishimura, Masaki Aikawa, Masaki Mori, Masami Matsumura

**Affiliations:** Division of General Internal Medicine, Jichi Medical University Hospital, 3311-1 Yakushiji, Shimotsuke, Tochigi 329-0498 Japan; Department of Internal Medicine, Division of Hematology, Jichi Medical University, Shimotsuke, Japan

**Keywords:** Diffuse large B-cell lymphoma, Anasarca, Edema, Hypothyroidism

## Abstract

**Background:**

Anasarca in patients with lymphoma is a rare symptom. We report a patient with DLBCL associated with pituitary gland infiltration that was diagnosed based on significant anasarca.

**Case presentation:**

A 72-year-old woman with a 10-year history of hypertension visited a local hospital presenting with anasarca and 15-kg weight gain in the past 3 months. we clinically diagnosed central hypothyroidism caused by pituitary gland infiltration of diffuse large B-cell lymphoma (DLBCL) (clinical stage IV in the Ann Arbor staging classification). The first course of chemotherapy improved anasarca remarkably and the patient’s body weight returned to what it was 3 months before.

**Conclusions:**

We experienced a patient with remarkable anasarca caused by DLBCL infiltration of the pituitary gland. A pituitary gland lesion with central hypothyroidism should be considered as one of the differential diagnoses of edema. This case was very valuable because we could assess it by following the time course of symptoms (edema and delayed relaxation time of the Achilles tendon reflex), laboratory data, and imaging findings (swelling anterior pituitary lobe).

## Background

Correct diagnosis is occasionally difficult in some patients with lymphoma. Typical symptoms of lymphoma, called B symptoms, include fever, night sweats, and weight loss. However, atypical manifestations are observed in some patients with lymphoma. Anasarca in patients with lymphoma is a rare symptom [1].

Diffuse large B-cell lymphoma (DLBCL) reveals extranodal involvements in more than 30% of patients at diagnosis [2,3]. The gastrointestinal tract, bone marrow, pancreas, liver, intravascular system, or brain is involved in patients with DLBCL. Some reports have shown the pituitary gland with extranodal involvement in patients with lymphoma [4,5].

We report a patient with DLBCL associated with pituitary gland infiltration that was diagnosed based on significant anasarca.

## Case presentation

A 72-year-old woman with a 10-year history of hypertension visited a local hospital presenting with anasarca and 15-kg weight gain in the past 3 months. She did not have fever, appetite loss, night sweats, or dyspnea. Because of elevated liver enzymes and abdominal computed tomography (CT) abnormalities, including splenomegaly and multiple splenic tumors, she was referred to our hospital.

On admission, physical examination of the patient revealed the following: temperature, 36.3 °C; pulse rate, 84 beats per minute, regular; blood pressure, 132/74 mmHg; and respiration rate, 18 breaths per minute. Although she had remarkable anasarca, she seemed in no distress. Her height was 143.5 cm, body weight 71 kg, and body mass index 34.2. She had no jugular venous distention. Lymphadenopathy was not noted. No crackles were audible, but there were diminished breath sounds in the base of the right lung. She had remarkable nonpitting and slow pitting mixed edema of the legs. A delayed relaxation time of the Achilles tendon reflex was observed.

**Figure 1:**
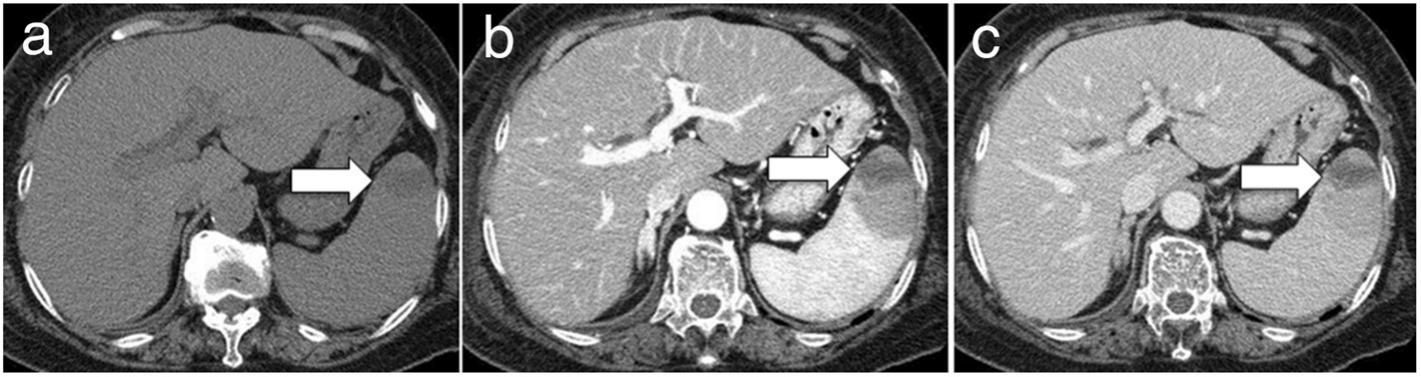
**Contrast-enhanced abdominal computed tomography image of the abdomen. (a)** Plain phase. **(b)** Arterial phase. **(c)** Equilibrium phase. Splenomegaly and multiple spleen tumors were observed. The maximal tumor size was 52 mm and the tumor had a necrotic lesion (white arrows).

**Figure 2:**
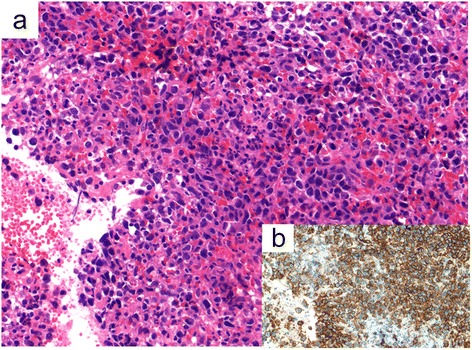
**Histopathology finding of the spleen. (a)** Hematoxylin-eosin stain. Aggregated large atypical cells were seen. The individual cells had chromatin-rich nuclei and relatively abundant intracytoplasmic eosinophilic inclusion bodies. **(b)** CD20 stain. Atypical cells were positive for CD20.

Laboratory test results revealed the following: white blood cell count, 7,400 cells/mm^3^; hemoglobin, 11.6 g/dL; platelet count, 103,000/μL; total protein, 6.0 g/dL; albumin, 2.9 g/dL; aspartate aminotransferase, 27 IU/L; alanine aminotransferase, 20 IU/L; lactate dehydrogenase, 707 IU/L; alkaline phosphatase, 629 IU/L; total-bilirubin, 0.69 mg/dL; blood urea nitrogen, 11 mg/dL; creatinine, 0.65 mg/dL; creatinine kinase, 15 IU/L; thyroid-stimulating hormone (TSH), 0.45 μU/mL (standard value: 0.45-3.33 μU/mL); free thyroxine (FT4), 0.37 ng/dL (standard value: 0.84-1.44 ng/dL); free triiodothyronine (FT3), 0.89 pg/mL (standard value: 2.11-3.51 pg/mL); and soluble interleukin-2 receptor, 6,660 U/mL (standard value: 124–466 U/mL). Urinary protein level was 0.2 g/g creatinine. A contrast-enhanced CT of the head, neck, chest, abdomen, and pelvis revealed splenomegaly and multiple spleen tumors (Figure 1), whereas there was no lymphadenopathy. CT-guided biopsy of the spleen was performed, which showed aggregated large atypical cells. The individual cells had the chromatin-rich nuclei and relatively abundant intracytoplasmic eosinophilic inclusion bodies (Figure 2). Immunohistochemistry showed that the atypical cells were positive for CD20 and CD79a, and negative for CD3 and CD10. Histopathology and immunohistochemistry of the spleen led to the diagnosis of DLBCL.

We assessed the central hypothyroidism because of anasarca, delayed Achilles tendon reflex, and low FT4.

**Figure 3:**
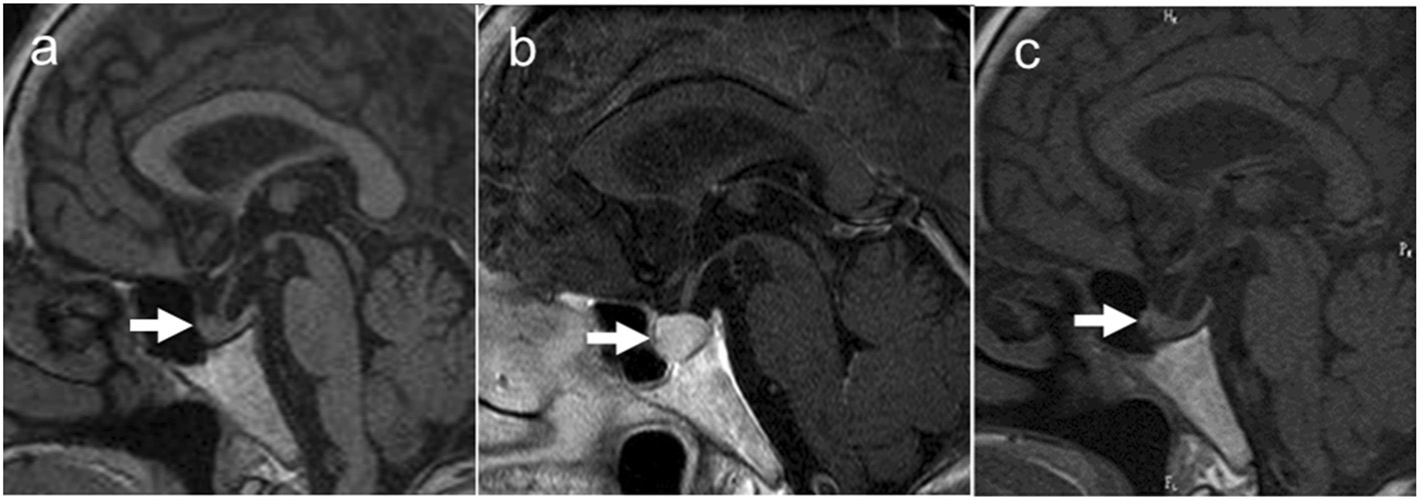
**Head magnetic resonance imaging.** White arrows indicate the pituitary gland. **(a)** One year before. Normal-sized pituitary gland. **(b)** Time of diagnosis. Pituitary gland swelling but keeping signal of posterior lobe of pituitary. **(c)** After one course of chemotherapy. Pituitary gland swelling improved.

Head magnetic resonance imaging (MRI) revealed swelling of pituitary gland, but a normal-size of pituitary gland was confirmed in a head MRI performed 1 year before (Figure 3a, b). Luteinizing hormone (LH) and follicle-stimulating hormone (FSH) were also at low levels (LH 0.3 mIU/mL [standard value: 6.7-38.0 mIU/mL] and FSH 3.4 mIU/mL [standard value: 26.2-113.3 mIU/mL]). Cortisol level was 21.6 μg/dL [standard value: 4.0-18.3μg/dL] and ACTH was 33.4 pg/mL [standard value: 7.2-63.3 pg/mL]. Prolactin was 9.2 ng/mL[standard value: 3.12-15.39 ng/mL(menopause women) ]. AVP level was not measured but there were no polyuria and hyponatremia. We diagnosed pituitary anterior lobe hormone insufficiency.

**Figure 4:**
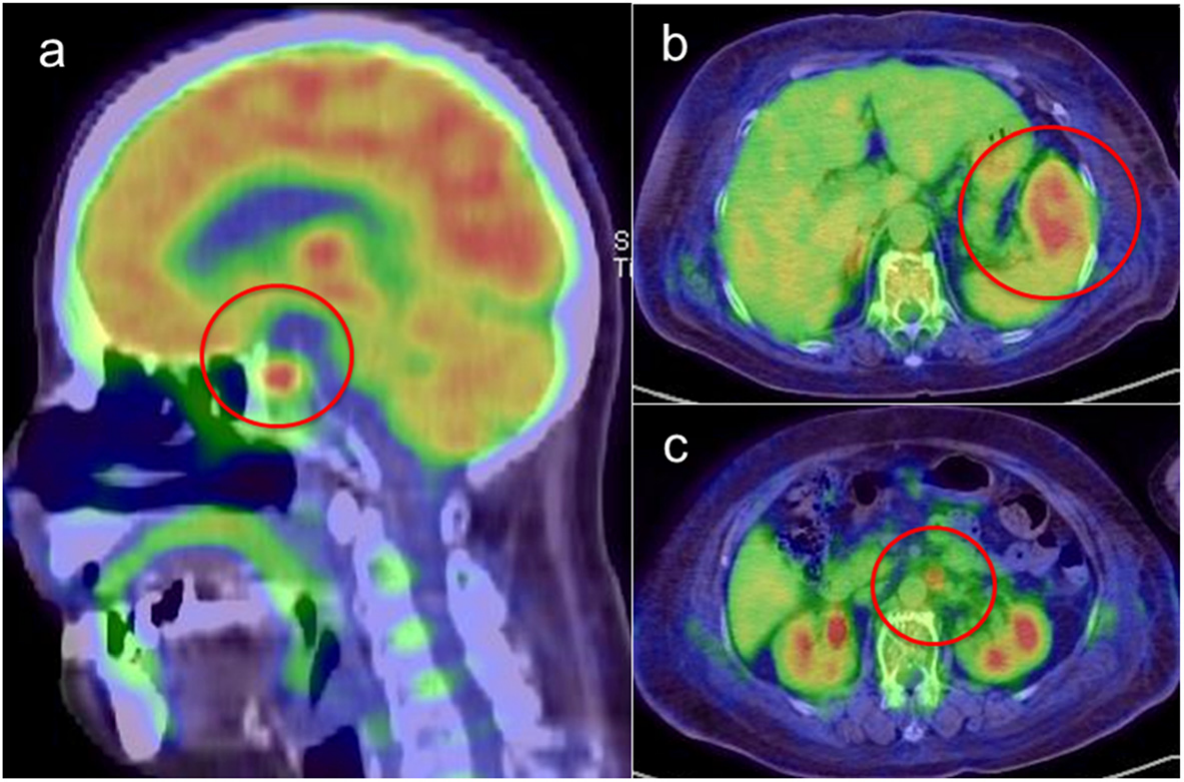
**Positron emission tomography (PET) scan.** PET scan revealed a localized accumulation in the pituitary gland **(a)**, spleen **(b)**, and para-abdominal aorta lymph nodes **(c)** (red circles).

Positron emission tomography (PET) scan revealed a localized accumulation in the pituitary gland, spleen, and para-abdominal aorta lymph nodes (Figure 4). Pituitary gland biopsy was not performed; however, we clinically diagnosed central hypothyroidism caused by pituitary gland infiltration of DLBCL (clinical stage IV in the Ann Arbor staging classification).

**Figure 5:**
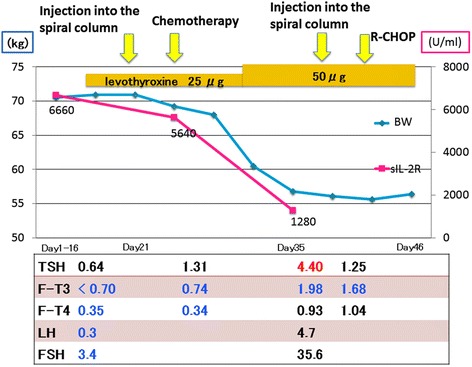
**Clinical course.** First course chemotherapy including methotrexate, vincristine, ifosfamide, and dexamethasone improved anasarca and body weight was reverted as same as 3 months before. Moreover, thyroid hormone, LH, and FSH became normal levels. Second course chemotherapy as cyclophosphamide, hydroxydaunorubicin, vincristine, and prednisone (CHOP) led to remission and the same regimen of chemotherapy was repeated.

She received 25 μg per day of levothyroxine for one week before the chemotherapy. However, the dose and duration of levothyroxine were not enough. The first course of chemotherapy for DLBCL including methotrexate, vincristine, ifosfamide, and dexamethasone, which improved anasarca remarkably and the patient’s body weight returned to what it was 3 months before. Moreover, thyroid hormone, LH, and FSH levels normalized and the pituitary gland swelling improved (Figure 3c), although thyroid hormone replacement therapy was not effective before chemotherapy (Figure 5). We chose a second course of chemotherapy that comprised cyclophosphamide, hydroxydaunorubicin, vincristine, and prednisone (CHOP) because there were no atypical cells in the patient’s cerebrospinal fluid. This second course of chemotherapy led to remission and the same chemotherapy regimen was repeated.

### Discussion

Here, we describe a case of pituitary gland infiltration of DLBCL associated with central hypothyroidism, which caused remarkable anasarca. In this case, only a few clinical manifestation were anasarca and a delayed relaxation time of the Achilles tendon reflex, other than the typical symptoms of lymphoma (fever, night sweats, and weight loss).

Pituitary gland involvement as extranodal lymphoma is comparatively rare [4,5], and DLBCL is one of the most frequent histological types for pituitary gland involvement [2]. Infiltration of lymphoma cells to the pituitary gland lead to headache, opthalmoplegia, or hemianopia [6], and often causes diabetes insipidus [6]. Anasarca is a rare symptom in patients with lymphoma [1].

Moreover, lymphoma with pituitary gland infiltration seldom shows significant anasarca or weight gain caused by secondary central hypothyroidism. Some patients with lymphoma who have pituitary gland involvement have no symptom, and they are incidentally detected using PET or MRI [7]. We clinically diagnosed infiltration of a malignant lymphoma in the pituitary gland based on time-dependent changes in MRI findings, thyroid hormone, LH, and FSH levels before and after chemotherapy; and PET findings.

Infiltration of DLBCL cells to the pituitary gland caused secondary central hypothyroidism, and secondary central hypothyroidism led to anasarca and weight gain in our patient. Nonpitting edema was noted, provably caused by hypothyroidism, and slow pitting edema resulting from hypoalbuminemia was observed simultaneously.

Elderly people often have several disorders at the same time; however, we believe that, in explaining manifestations, one disorder should account for every symptom. In this case, the patient had remarkable anasarca because of DLBCL involvement of the pituitary gland. This case was very valuable because we could assess the patient’s abnormalities; that is to say, anasarca, weight gain, hypothyroidism, elevated lactate dehydrogenase and alkaline phosphatase, splenomegaly, and swelling of the pituitary gland caused by malignant lymphoma and its infiltration into the pituitary gland.

In this era of longevity, the prevalence of diseases increases, and we must anticipate the greater likelihood of multiple, simultaneous diagnoses [8]. Hickam’s dictum and Occam’s razor are well suited to this case. “A patient can have as many diagnoses as he darn well pleases” [8]. This is Hickam’s dictum. However, William of Ockham stated “Among competing hypotheses, favor the simplest one” [8]. This is known as Occam’s razor.

It was easy to assess laboratory abnormalities, splenomegaly, and multiple spleen tumors that resulted from the malignant lymphoma. It was also easy to assess anasarca that was due to hypothyroidism and hypoalbuminemia. In this case, however, we reached the correct diagnosis of pituitary gland involvement from DLBCL, which manifested significant anasarca, according to Occam’s razor. A pituitary gland lesion with central hypothyroidism should be considered as one of the differential diagnoses of edema, especially nonpitting edema in some cases.

## Conclusion

We experienced a patient with remarkable anasarca caused by DLBCL infiltration of the pituitary gland. A pituitary gland lesion with central hypothyroidism should be considered as one of the differential diagnoses of edema. This case was very valuable because we could assess it by following the time course of symptoms, laboratory data, and imaging findings.

### Consent

Written informed consent was obtained from the patient for publication of this Case report and any accompanying images.

A copy of the written consent is available for review by the Series Editor of this journal.
